# Supplementary material for: Structural basis for the tryptophan sensitivity of TnaC-mediated ribosome stalling
Source: Nat Commun. 2021 Sep 9;12:5340. doi: 10.1038/s41467-021-25663-8 (PMC8429421; doi:10.1038/s41467-021-25663-8)
Supplement: Supplementary file 1 — Supplementary Information [file 41467_2021_25663_MOESM1_ESM.pdf]

SUPPLEMENTARY INFORMATION FOR

# Structural basis for the tryptophan sensitivity of TnaC-mediated ribosome stalling

Anne-Xander van der Stel[1], Emily R. Gordon[2], Arnab Sengupta[2], Allyson K. Martínez[3], Dorota Klepacki[4], Thomas N. Perry[1], Alba Herrero del Valle[1], Nora Vázquez-Laslop[4], Matthew S. Sachs[3], Luis R. Cruz-Vera[2] & C. Axel Innis[1]

[1] Univ. Bordeaux, Centre National de la Recherche Scientifique, Institut National de la Santé et de la Recherche Médicale, ARNA, UMR 5320, U1212, Institut Européen de Chimie et Biologie, F-33600 Pessac, France.

[2] Department of Biological Sciences, University of Alabama in Huntsville, Huntsville, AL 35899, USA.

[3] Department of Biology, Texas A&M University, College Station, TX 77843, USA.

[4] Center for Biomolecular Sciences, University of Illinois at Chicago, Chicago, IL 60607, USA.

## SUPPLEMENTARY NOTES

### Isolation of suppressors of the loss-of-function mutant *tnaC* (D16E) gene

Intragenic suppressor mutations to the loss-of-function *tnaC* D16E gene were obtained using the strain AW845 (MG1655 Δ7 *rrn* Δ(*lacZYA*) Δ(*tnaCAB*) *att7*::$tna_p$*tnaC*(*D16E*)(*tnaA'*-'*lacZYA*) (p*rrnC*-*sacB*, ptRNA67), which carries a *tnaA–lacZ* protein fusion whose expression is under the control of the *tna* operon regulatory leader region (Ref. 16). This bacterial strain lacks tryptophanase activity, which as a consequence facilitates experimental manipulation of internal L-Trp concentration through addition of exogenous L-Trp. Bacterial cultures were grown overnight in Vogel Bonner (VB) minimal medium supplemented with 0.2% glucose, 0.05% acid-hydrolyzed casein, 0.01% vitamin B1 and 25 μg/ml kanamycin. Cells were washed once in VB without any supplements and 100 μl of washed cells were plated on VB minimal medium supplemented with 1% lactose, 100 μg/ml L-Trp, 0.01% vitamin B1 and 25 μg/ml kanamycin. Plates were incubated at 37°C for 6 days. Colonies that formed were picked and each colony was grown overnight in VB minimal medium supplemented with 0.2% glucose, 0.05% acid-hydrolyzed casein, 0.01% vitamin B1 and 25 μg/ml kanamycin. Since mutations that restore induction to the uninducible TnaC(D16E) mutant were desired, these cultures were screened for L-Trp inducibility. Overnight cultures were replica plated onto VB plates supplemented with 1% lactose, 0.01% vitamin B1, and 25 μg/ml kanamycin with or without 100 μg/ml Trp. Colonies that either grew on the L-Trp containing plate and not at all on the plate lacking L-Trp, or those that grew better on the L-Trp containing plate than they did on the plate lacking L-Trp were selected for further analysis by standard Miller Assay. Out of the 1,152 total colonies screened by replica plating, 111 showing differential growth were analyzed by Miller Assay of exponentially growing cultures to determine if the LacZ reporter was inducible by L-Trp. Out of these 111 colonies, 13 were confirmed to be inducible by L-Trp in three independent experiments. The leader region of the reporter operon from the 13 mutants was sequenced to determine if there were any mutations within *tnaC* using primers listed in Supplementary Table 3. Based on the *tnaC* sequence, the mutants were divided into 4 classes. Nearly half of the mutants were true revertants, which means that the combined selection and screening method is a powerful tool that can be used to identify mutations that restore L-Trp-inducibility to the non-functional D16E mutant, or in principle any other non-functional TnaC mutant. Mutations at two different non-conserved codon positions within *tnaC* were found to suppress the non-functional D16E replacement. To ensure that the second-site mutations within *tnaC* were responsible for restoring L-Trp-mediated ribosome arrest, strains containing reporter operons with double and single mutational changes in *tnaC* were constructed in a MG1655 Δ(*lacZYA*) strain (Ref. 16), using the plasmids and primers indicated in Supplementary Table 3. Derivatives lacking an AUG start codon were also constructed to determine whether the effects of these mutations on the expression of the reporter genes were dependent on the translation of the *tnaC* gene variants. Expression levels of all these constructs are shown in Supplementary Table1.

### Determination of the importance of the amino terminal end of TnaC on ribosome stalling

To determine the minimal elements of the TnaC peptide required for ribosome stalling, we performed *in vitro* coupled transcription-translation assays (Ref. 16) using several *tnaC* constructs obtained by PCR that differ in the 5′-end of their open reading frame, generating N-terminally truncated versions of the TnaC peptide (Supplementary Fig. 6a). Accumulation of [$^{35}$S]-TnaC-tRNA$^{Pro}$, an indicator of ribosome stalling, was determined by resolving the reaction products in 10% Tris-tricine electrophoresis gels (Ref. 16). We observed that deleting residues 2-8 does not affect the accumulation of [$^{35}$S]-TnaC-tRNA$^{Pro}$ at higher L-Trp concentrations (5 mM), but results in

a peptide that is less sensitive to lower concentrations of L-Trp (Supplementary Fig. 6b, compare data for TnaC-16 with TnaC-24). However, deletion of residues 2–10 abolished the accumulation of [$^{35}$S]-TnaC-tRNA$^{Pro}$ (Supplementary Fig. 6b, compare data for TnaC-15 with TnaC-24). These results were corroborated using toeprinting assays (Ref. 16), which showed that deletion of residues 2–9 still produces a functional TnaC peptide at higher concentrations of L-Trp (Supplementary Fig. 6c, compare data for TnaC-15 with TnaC-24). None of the shorter peptides were functional (Supplementary Fig. 6c, compare data for TnaC-14 through TnaC-7 with TnaC-24). Overall, these results indicate that residues 10–24 contain enough structural information to detect L-Trp and inhibit translation termination.

**Table S1 | R23H is a *cis*-acting mutation that suppresses the loss-of-function D16E mutation**

| Strain | β-galactosidase activity (MU)[a] | | Induction ratio (+Trp/–Trp)[b] |
|---|---|---|---|
| | –Trp | +Trp | |
| Wild-Type | 57 ± 2 | 2175 ± 274 | 38.2 |
| D16E | 38 ± 11 | 68 ± 25 | 1.8 |
| R23H | 1147 ± 40 | 1636 ± 317 | 1.4 |
| D16E/R23H | 192 ± 32 | 453 ± 10 | 2.4 |
| Wild-type (ΔAUG)[c] | 7 ± 1 | 7 ± 0 | 1.0 |
| D16E (ΔAUG) | 7 ± 1 | 7 ± 0 | 1.0 |
| R23H (ΔAUG) | 5 ± 0 | 5 ± 0 | 1.0 |
| D16E/R23H (ΔAUG) | 6 ± 1 | 5 ± 0 | 0.8 |

[a] Cultures of *rrn+ E. coli* bacterial strains AW153 (Wild-type), AW643 (Wild-type(ΔAUG)), AW821 (D16E), AW922 (R23H), AW925 (D16E/R23H), AW946 (D16E (ΔAUG)), AW961 (R23H (ΔAUG)), and AW973 (D16E/R23H (ΔAUG)) were grown in minimal medium supplemented with 0.2% glycerol, 0.05% acid-hydrolyzed casein, 0.01% vitamin B1, with 100 μg/ml additional Trp (+Trp) or without additional Trp (–Trp). β-Galactosidase assays were performed in three independent experiments.

[b] Ratio of values for cultures grown with (+Trp) or without (–Trp) additional Trp.

[c] The start codon of *tnaC* was replaced with a UAG stop codon. Absence of expression of β-galactosidase in these constructs indicates that translation of *tnaC* is required for the L-Trp induced expression of the reporter gene.

# Table S2 | Cryo-EM statistics

| Data collection and processing | | | | |
|---|---|---|---|---|
| Dataset | TnaC | TnaC(R23F)_A | TnaC(R23F)_B | |
| Reconstruction | TnaC–70S | TnaC(R23F)–70S | | TnaC(R23F)–70S–RF2 |
| **Magnification** | | | | |
| Voltage (kV) | 200 | 300 | 200 | |
| Electron exposure ($e^-$/ Å$^2$) | 40 | 44 | 40 | |
| Defocus range (μm) | 1.0 to 2.0 | 0.4 to 1.6 | 0.4 to 2.0 | |
| Pixel size (Å) | 0.93 | 0.827 | 0.93 | 0.93 |
| Symmetry imposed | C1 | C1 | | C1 |
| Initial particle images (no.) | 905,648 | 358,000 | 1,381,950 | |
| Final particle images (no.) | 93,588 | 191,230 | | 113,840 |
| Map resolution (Å) | 2.87 | 2.41 | | 2.62 |
| FSC threshold | 0.143 | 0.143 | | 0.143 |
| Map resolution range (Å) | 2.6-12.0 | 2.1-6.0 | | 2.4-4.9 |

| Refinement | | | | |
|---|---|---|---|---|
| Initial model used (PDB code) | 6TBV | 6TBV | | TnaC(R23F)–70S (RF2: 6OUO) |
| Model resolution (Å) | 2.7 | 2.7 | | 2.4 |
| FSC threshold | 0.143 | 0.143 | | 0.143 |
| Model resolution range (Å) | 2.5-8.7 | 2.5-8.7 | | 2.1-6.0 |
| Map sharpening B factor (Å$^2$) | -59 | -27 | | -34 |
| **Model composition** | | | | |
| Non-hydrogen atoms | 145,039 | 146,611 | | 148,251 |
| Protein residues | 5,691 | 5,691 | | 6,048 |
| RNA bases | 4,638 | 4,640 | | 4,641 |
| Ligands | 175 | 1,702 | | 489 |
| **B-factor (Å$^2$)** | | | | |
| Protein | 42.3 | 46.2 | | 48.2 |
| Ligand | 30.1 | 26.7 | | 48.8 |
| **R.m.s. deviations** | | | | |
| Bond lengths (Å) | 0.010 | 0.006 | | 0.013 |
| Bond angles (º) | 0.922 | 0.980 | | 1.05 |
| **Validation** | | | | |
| Model to map CC (main chain) | 0.82 | 0.82 | | 0.79 |
| Model to map CC (side chain) | 0.85 | 0.85 | | 0.83 |
| MolProbity score | 1.62 | 1.54 | | 1.73 |
| Clashscore | 6.55 | 5.4 | | 6.84 |
| Poor rotamers (%) | 0.04 | 0.06 | | 0.02 |
| **Ramachandran plot** | | | | |
| Favored (%) | 96.22 | 96.28 | | 94.81 |
| Allowed (%) | 3.71 | 3.70 | | 5.10 |
| Disallowed (%) | 0.07 | 0.02 | | 0.08 |

## Table S3 | Constructs and oligonucleotides

| Name | Description | Sequence (5′ to 3′) | Supplier | Reference |
|---|---|---|---|---|
| Att7F | Oligo used to PCR amplify the leader region of Tna-LacZ reporter genes | GCGGCGACAACAGTTGCGACGGTGGTACG | | Ref 16 |
| AW111 | Oligo used to PCR amplify the leader region of Tna-LacZ reporter genes | GCGGTTTCTCCGGCGCGTAAAAATGCGCTCAGG | | Ref 16 |
| M13F-200 | Oligo to sequence PCR products of the leader region of Tna-LacZ reporter genes | CCATTCGCCATTCAGGCTGCGCAAC | | Ref 16 |
| pAW627 | pUC18 Plasmid based used to generate *tnaC* R23H mutants and to insert Tna-LacZ reporter genes at the *att* site of *E. coli* chromosome. Codons N2 through H22 are replaced by a BsaI-XhoI-BsaI linker. | TCATTGTTACCACTCCTGTTATTCCTCAACCCTTTTTTTAAACATTAAAATTCTTACGTAATTTATAATCTTTAAAAAAAGCATTTAATATTGCTCCCCGAACGATTGTGATTCGATTCACATTTAAACAATTTCAGAATAGACAAAAACTCTGAGTGTAATAATGTAGCCTCGTGTCTTGCGAGGATAAGTGCATTATGAATGGTCTCTCGAGGGTCTCGCCCTTGATTTGCCCTTCTGTAGCCATCACCAGAGCCAAACCGATTAGATTCAATGTGATCTATTTGTTTGCTATATCTTAATTTTGCCTTTTGCAAAGGTCATCTCTCGTTTATTTACTTGTTTTAGTAAATGATGGTGCTTGCATATATATCTGGCGAATTAATCGGTATAGCAGATGTAATATTCACAGGGATCACTGTAATTAAAATAAATGAAGGATTATG | | This study |
| pAW629 | pUC18 Plasmid based used to generate *tnaC* start-stop codon mutants and to insert Tna-LacZ reporter genes at the *att* site of *E. coli* chromosome. TnaC start codon is replaced by a TGA stop codon | TCATTGTTACCACTCCTGTTATTCCTCAACCCTTTTTTTAAACATTAAAATTCTTACGTAATTTATAATCTTTAAAAAAAGCATTTAATATTGCTCCCCGAACGATTGTGATTCGATTCACATTTAAACAATTTCAGAATAGACAAAAACTCTGAGTGTAATAATGTAGCCTCGTGTCTTGCGAGGATAAGTGCATTTGAAATATCTTACATATATGTGTGACCTCAAAATGGTTCAATATTGACAACAAAATTGTCGATCACCGCCCTTGATTTGCCCTTCTGTAGCCATCACCAGAGCCAAACCGATTAGATTCAATGTGATCTATTTGTTTGCTATATCTTAATTTTGCCTTTTGCAAAGGTCATCTCTCGTTTATTTACTTGTTTTAGTAAATGATGGTGCTTGCATATATATCTGGCGAATTAATCGGTATAGCAGATGTAATATTCACAGGGATCACTGTAATTAAAATAAATGAAGGATTATG | | This study |
| AW303 | Oligo used to generate tnaC R23H at the pAW627 and pAW629 plasmid | GTCGATCACCACCCTTGATTTGCCCTTCTG | Eurofins, USA | This study |

| | | | | |
|---|---|---|---|---|
| AW304 | Oligo used to generate tnaC R23H at the pAW627 and pAW629 plasmid | CAGAAGGGCAAATCAAGGGTGGTGATC GAC | Eurofins, USA | This study |
| AW305 | Oligo used to generate tnaC R23H D16Eat the pAW627 and pAW629 plasmid | GGTTTAATATTGAAAACAAGATTGTCGAT C | Eurofins, USA | This study |
| AW306 | Oligo used to generate tnaC R23H D16E at pAW627 and pAW629 plasmid | GATCGACAATCTTGTTTTCAATATTAAACC | Eurofins, USA | This study |
| pGF2500 | Template for preparation of wild type tnaC PCR amplifications | TCATTGTTACCACTCCTGTTATTCCTCAAC CCTTTTTTTAAACATTAAAATTCTTACGTAA TTTATAATCTTTAAAAAAAGCATTTAATATT GCTCCCCGAACGATTGTGATTCGATTCAC ATTTAAACAATTTCAGAATAGACAAAAAC TCTGAGTGTAATAATGTAGCCTCGTGTCT TGCGAGGATAAGTGCATTATGAATATCTT ACATATATGTGTGACCTCAAAATGGTTCA ATATTGACAACAAAATTGTCGATCACCGC CCTTGATTTGCCCTTCTGTAGCCATCACC AGAGCCAAACCGATTAGATTCAATGTGAT CTATTTGTTTGCTATATCTTAATTTTGCCTT TTGCAAAGGTCATCTCTCGTTTATTTACTT GTTTTAGTAAATGATGGTGCTTGCATATA TATCTGGCGAATTAATCGGTATAGCAGAT GTAATATTCACAGGGATCACTGTAATTAA AATAAATGAAGGATTATG | | Ref 8 |
| pGF2414 | Template for preparation of W12R tnaC PCR amplifications | TCATTGTTACCACTCCTGTTATTCCTCAAC CCTTTTTTTAAACATTAAAATTCTTACGTAA TTTATAATCTTTAAAAAAAGCATTTAATATT GCTCCCCGAACGATTGTGATTCGATTCAC ATTTAAACAATTTCAGAATAGACAAAAAC TCTGAGTGTAATAATGTAGCCTCGTGTCT TGCGAGGATAAGTGCATTATGAATATCTT ACATATATGTGTGACCTCAAAACGCTTCA ATATTGACAACAAAATTGTCGATCACCGC CCTTGATTTGCCCTTCTGTAGCCATCACC AGAGCCAAACCGATTAGATTCAATGTGAT CTATTTGTTTGCTATATCTTAATTTTGCCTT TTGCAAAGGTCATCTCTCGTTTATTTACTT GTTTTAGTAAATGATGGTGCTTGCATATA TATCTGGCGAATTAATCGGTATAGCAGAT GTAATATTCACAGGGATCACTGTAATTAA AATAAATGAAGGATTATG | | Ref 8 |
| pGF2500 R23F | Template for preparation of tnaC R23F PCR amplifications | TCATTGTTACCACTCCTGTTATTCCTCAAC CCTTTTTTTAAACATTAAAATTCTTACGTAA TTTATAATCTTTAAAAAAAGCATTTAATATT GCTCCCCGAACGATTGTGATTCGATTCAC ATTTAAACAATTTCAGAATAGACAAAAAC TCTGAGTGTAATAATGTAGCCTCGTGTCT | | This study |

| | | | |
|---|---|---|---|
| | | TGCGAGGATAAGTGCATTATGAATATCTT<br>ACATATATGTGTGACCTCAAAATGGTTCA<br>ATATTGACAACAAAATTGTCGATCACTTC<br>CCTTGATTTGCCCTTCTGTAGCCATCACC<br>AGAGCCAAACCGATTAGATTCAATGTGAT<br>CTATTTGTTTGCTATATCTTAATTTTGCCTT<br>TTGCAAAGGTCATCTCTCGTTTATTTACTT<br>GTTTTAGTAAATGATGGTGCTTGCATATA<br>TATCTGGCGAATTAATCGGTATAGCAGAT<br>GTAATATTCACAGGGATCACTGTAATTAA<br>AATAAATGAAGGATTATG | |
| pGF2500 W12R R23F | Template for preparation of tnaC W12R R23F PCR amplifications | TCATTGTTACCACTCCTGTTATTCCTCAAC<br>CCTTTTTTTAAACATTAAAATTCTTACGTAA<br>TTTATAATCTTTAAAAAAAGCATTTAATATT<br>GCTCCCCGAACGATTGTGATTCGATTCAC<br>ATTTAAACAATTTCAGAATAGACAAAAAC<br>TCTGAGTGTAATAATGTAGCCTCGTGTCT<br>TGCGAGGATAAGTGCATTATGAATATCTT<br>ACATATATGTGTGACCTCAAAACGCTTCA<br>ATATTGACAACAAAATTGTCGATCACTTC<br>CCTTGATTTGCCCTTCTGTAGCCATCACC<br>AGAGCCAAACCGATTAGATTCAATGTGAT<br>CTATTTGTTTGCTATATCTTAATTTTGCCTT<br>TTGCAAAGGTCATCTCTCGTTTATTTACTT<br>GTTTTAGTAAATGATGGTGCTTGCATATA<br>TATCTGGCGAATTAATCGGTATAGCAGAT<br>GTAATATTCACAGGGATCACTGTAATTAA<br>AATAAATGAAGGATTATG | This study |
| pGF2500 ^25R | Template for preparation of tnaC ^25R PCR amplifications | TCATTGTTACCACTCCTGTTATTCCTCAAC<br>CCTTTTTTTAAACATTAAAATTCTTACGTAA<br>TTTATAATCTTTAAAAAAAGCATTTAATATT<br>GCTCCCCGAACGATTGTGATTCGATTCAC<br>ATTTAAACAATTTCAGAATAGACAAAAAC<br>TCTGAGTGTAATAATGTAGCCTCGTGTCT<br>TGCGAGGATAAGTGCATTATGAATATCTT<br>ACATATATGTGTGACCTCAAAATGGTTCA<br>ATATTGACAACAAAATTGTCGATCACCGC<br>CCTCGCTGATTTGCCCTTCTGTAGCCATC<br>ACCAGAGCCAAACCGATTAGATTCAATGT<br>GATCTATTTGTTTGCTATATCTTAATTTTGC<br>CTTTTGCAAAGGTCATCTCTCGTTTATTTA<br>CTTGTTTTAGTAAATGATGGTGCTTGCAT<br>ATATATCTGGCGAATTAATCGGTATAGCA<br>GATGTAATATTCACAGGGATCACTGTAAT<br>TAAAATAAATGAAGGATTATG | This study |
| pGF2500 W12R ^25R | Template for preparation of tnaC W12R ^25R PCR amplifications | TCATTGTTACCACTCCTGTTATTCCTCAAC<br>CCTTTTTTTAAACATTAAAATTCTTACGTAA<br>TTTATAATCTTTAAAAAAAGCATTTAATATT<br>GCTCCCCGAACGATTGTGATTCGATTCAC<br>ATTTAAACAATTTCAGAATAGACAAAAAC<br>TCTGAGTGTAATAATGTAGCCTCGTGTCT<br>TGCGAGGATAAGTGCATTATGAATATCTT<br>ACATATATGTGTGACCTCAAAACGCTTCA<br>ATATTGACAACAAAATTGTCGATCACCGC<br>CCTCGCTGATTTGCCCTTCTGTAGCCATC<br>ACCAGAGCCAAACCGATTAGATTCAATGT<br>GATCTATTTGTTTGCTATATCTTAATTTTGC<br>CTTTTGCAAAGGTCATCTCTCGTTTATTTA | This study |

| | | | | |
|---|---|---|---|---|
| | | CTTGTTTTAGTAAATGATGGTGCTTGCAT ATATATCTGGCGAATTAATCGGTATAGCA GATGTAATATTCACAGGGATCACTGTAAT TAAAATAAATGAAGGATTATG | | |
| pGF2500 R23F ^25R | Template for preparation of tnaC R23F ^25R PCR amplifications | TCATTGTTACCACTCCTGTTATTCCTCAAC CCTTTTTTTAAACATTAAAATTCTTACGTAA TTTATAATCTTTAAAAAAAGCATTTAATATT GCTCCCCGAACGATTGTGATTCGATTCAC ATTTAAACAATTTCAGAATAGACAAAAAC TCTGAGTGTAATAATGTAGCCTCGTGTCT TGCGAGGATAAGTGCATTATGAATATCTT ACATATATGTGTGACCTCAAAATGGTTCA ATATTGACAACAAAATTGTCGATCACTTC CCTCGCTGATTTGCCCTTCTGTAGCCATC ACCAGAGCCAAACCGATTAGATTCAATGT GATCTATTTGTTTGCTATATCTTAATTTTGC CTTTTGCAAAGGTCATCTCTCGTTTATTTA CTTGTTTTAGTAAATGATGGTGCTTGCAT ATATATCTGGCGAATTAATCGGTATAGCA GATGTAATATTCACAGGGATCACTGTAAT TAAAATAAATGAAGGATTATG | | This study |
| pGF2500 W12R R23F ^25R | Template for preparation of tnaC W12R R23F ^25R PCR amplifications | TCATTGTTACCACTCCTGTTATTCCTCAAC CCTTTTTTTAAACATTAAAATTCTTACGTAA TTTATAATCTTTAAAAAAAGCATTTAATATT GCTCCCCGAACGATTGTGATTCGATTCAC ATTTAAACAATTTCAGAATAGACAAAAAC TCTGAGTGTAATAATGTAGCCTCGTGTCT TGCGAGGATAAGTGCATTATGAATATCTT ACATATATGTGTGACCTCAAAACGCTTCA ATATTGACAACAAAATTGTCGATCACTTC CCTCGCTGATTTGCCCTTCTGTAGCCATC ACCAGAGCCAAACCGATTAGATTCAATGT GATCTATTTGTTTGCTATATCTTAATTTTGC CTTTTGCAAAGGTCATCTCTCGTTTATTTA CTTGTTTTAGTAAATGATGGTGCTTGCAT ATATATCTGGCGAATTAATCGGTATAGCA GATGTAATATTCACAGGGATCACTGTAAT TAAAATAAATGAAGGATTATG | | This study |
| T7-tnaC24 forward | Oligo to produce PCR fragments used in toe-printing assays | TAATACGACTCACTATAGGGAGTTTTATA AGGAGGAAAACATATGAATATCTTACATA TATGT | Eurofins, USA | Ref 16 |
| tnaC reverse toe | Oligo to produce PCR fragments used in toe-printing assays | AGCAAACAAATAGATCACATTG | Eurofins, USA | Ref 16 |
| RpoC-ECOR | Oligo to produce PCR fragments used to generate mRNA | ACGGAATTCCTTGCCGAGTTTGACTC | Eurofins, USA | Ref 8 |
| pEX28-tnaCWT | Template for Cryo-EM complex preparation, biscistronic operon of tnacWT | CGATCGAATTCTAATACGACTCACTATAG GGCTTAAGTATAAGGAGGAAAAAATATG AATATCTTACATATATGTGTGACCTCAAAA TGGTTCAATATTGACAACAAAATTGTCGA TCACCGCCCGTGATTTGCCCTTCTGTAGC CACATCCAGAGCCAAGTTTTATAAGGAG GAAAAAATATGAATATCTTACATATATGT GTGACCTCAAAATGGTTCAATATTGACAA | Eurofins | This study |

| | | | | |
|---|---|---|---|---|
| | | CAAAATTGTCGATCACCGCCCGTGATTTG CCCTTCTGTAGCCATCACCAGAGCCAGAT ATCTAGCATAACCCCTTGGGGCCTCTAAA CGGGTCTTGAGGGGTTTTTTG | | |
| pEX28-tnaCR23F | Template for Cryo-EM complex preparation, biscistronic operon of tnaC-(R23F) | CGATCGAATTCTAATACGACTCACTATAG GGCTTAAGTATAAGGAGGAAAAAATATG AATATCTTACATATATGTGTGACCTCAAAA TGGTTCAATATTGACAACAAAATTGTCGA TCACTTTCCGTGATTTGCCCTTCTGTAGCC ACATCCAGAGCCAAGTTTTATAAGGAGG AAAAAATATGAATATCTTACATATATGTGT GACCTCAAAATGGTTCAATATTGACAACA AAATTGTCGATCACTTTCCGTGATTTGCC CTTCTGTAGCCATCACCAGAGCCAGATAT CTAGCATAACCCCTTGGGGCCTCTAAAC GGGTCTTGAGGGGTTTTTTG | Eurofins | This study |
| RNaseH oligo | Oligo to direct RNaseH to cut between the two cistron during Cryo-EM sample preparation | CCTCCTTATAAAACT | Eurogentec | Ref. 26 |
| pETDuet prfB wt | Template for expression of wild type RF2 protein | AAAAUCUCCGGCGAUUACGCUUACGG CUGGCUGCGUACAGAAACCGGCGUUC ACCGCCUGGUGCGUAAAAGCCCGUUU GACUCCGGCGGUCGUCGCCACACGUC GUUCAGCUCCGCGUUUGUUUAUCCGG AAGUUGAUGAUGAUAUUGAUAUCGAA AUCAACCCGGCGGAUCUGCGCAUUGA CGUUUAUCGCACGUCCGGCGCGGGCG GUCAGCACGUUAACCGUACCGAAUCU GCGGUGCGUAUUACCCACAUCCCGACC GGGAUCGUGACCCAGUGCCAGAACGA CCGUUCCCAGCACAAGAACAAAGAUCA GGCCAUGAAGCAGAU | | Ref. 24 |
| pETDuet prfB Q252A | Template for expression of RF2 Q252A mutant protein | AAAAUCUCCGGCGAUUACGCUUACGG CUGGCUGCGUACAGAAACCGGCGUUC ACCGCCUGGUGCGUAAAAGCCCGUUU GACUCCGGCGGUCGUCGCCACACGUC GUUCAGCUCCGCGUUUGUUUAUCCGG AAGUUGAUGAUGAUAUUGAUAUCGAA AUCAACCCGGCGGAUCUGCGCAUUGA CGUUUAUCGCACGUCCGGCGCGGGCG GUGCGCACGUUAACCGUACCGAAUCU GCGGUGCGUAUUACCCACAUCCCGACC GGGAUCGUGACCCAGUGCCAGAACGA CCGUUCCCAGCACAAGAACAAAGAUCA GGCCAUGAAGCAGAU | | This study |
| pETDuet prfB S205P | Template for expression of RF2 S205P mutant protein | AAAAUCUCCGGCGAUUACGCUUACGG CUGGCUGCGUACAGAAACCGGCGUUC ACCGCCUGGUGCGUAAACCCCCGUUU GACUCCGGCGGUCGUCGCCACACGUC GUUCAGCUCCGCGUUUGUUUAUCCGG AAGUUGAUGAUGAUAUUGAUAUCGAA AUCAACCCGGCGGAUCUGCGCAUUGA CGUUUAUCGCACGUCCGGCGCGGGCG GUCAGCACGUUAACCGUACCGAAUCU GCGGUGCGUAUUACCCACAUCCCGACC | | This study |

| | | | | |
|---|---|---|---|---|
| | | GGGAUCGUGACCCAGUGCCAGAACGACCGUUCCCAGCACAAGAACAAAGAUCAGGCCAUGAAGCAGAU | | |
| $tRNA_2^{Pro}$ primer | Oligo used in Northern blots to detect $tRNA_2^{Pro}$-containg molecules | CCCTAGTTTAAGGCC | Eurofins, USA | Ref. 29 |
| T7-tnaC22 forward | Oligo to produce PCR fragments used in toe-printing assays | TAATACGACTCACTATAGGGAGTTTTATAAGGAGGAAAACATATGTTACATATATGTGTGACC | Eurofins, USA | This study |
| T7-tnaC20 forward | Oligo to produce PCR fragments used in toe-printing assays | TAATACGACTCACTATAGGGAGTTTTATAAGGAGGAAAACATATGATATGTGTGACCTCAAAA | Eurofins, USA | This study |
| T7-tnaC18 forward | Oligo to produce PCR fragments used in toe-printing assays | TAATACGACTCACTATAGGGAGTTTTATAAGGAGGAAAACATATGGTGACCTCAAAATGGTTC | Eurofins, USA | This study |
| T7-tnaC16 forward | Oligo to produce PCR fragments used in toe-printing assays | TAATACGACTCACTATAGGGAGTTTTATAAGGAGGAAAACATATGTCAAAATGGTTCAATATT | Eurofins, USA | This study |
| T7-tnaC15 forward | Oligo to produce PCR fragments used in toe-printing assays | TAATACGACTCACTATAGGGAGTTTTATAAGGAGGAAAACATATGAAATGGTTCAATATTGAC | Eurofins, USA | This study |
| T7-tnaC14 forward | Oligo to produce PCR fragments used in toe-printing assays | TAATACGACTCACTATAGGGAGTTTTATAAGGAGGAAAACATATGTGGTTCAATATTGACAAC | Eurofins, USA | This study |
| T7-tnaC13 forward | Oligo to produce PCR fragments used in toe-printing assays | TAATACGACTCACTATAGGGAGTTTTATAAGGAGGAAAACATATGTTCAATATTGACAACAAA | Eurofins, USA | This study |
| T7-tnaC11 forward | Oligo to produce PCR fragments used in toe-printing assays | TAATACGACTCACTATAGGGAGTTTTATAAGGAGGAAAACATATGATTGACAACAAAATTGTC | Eurofins, USA | This study |
| T7-tnaC9 forward | Oligo to produce PCR fragments used in toe-printing assays | TAATACGACTCACTATAGGGAGTTTTATAAGGAGGAAAACATATGAACAAAATTGTCGATCAC | Eurofins, USA | This study |

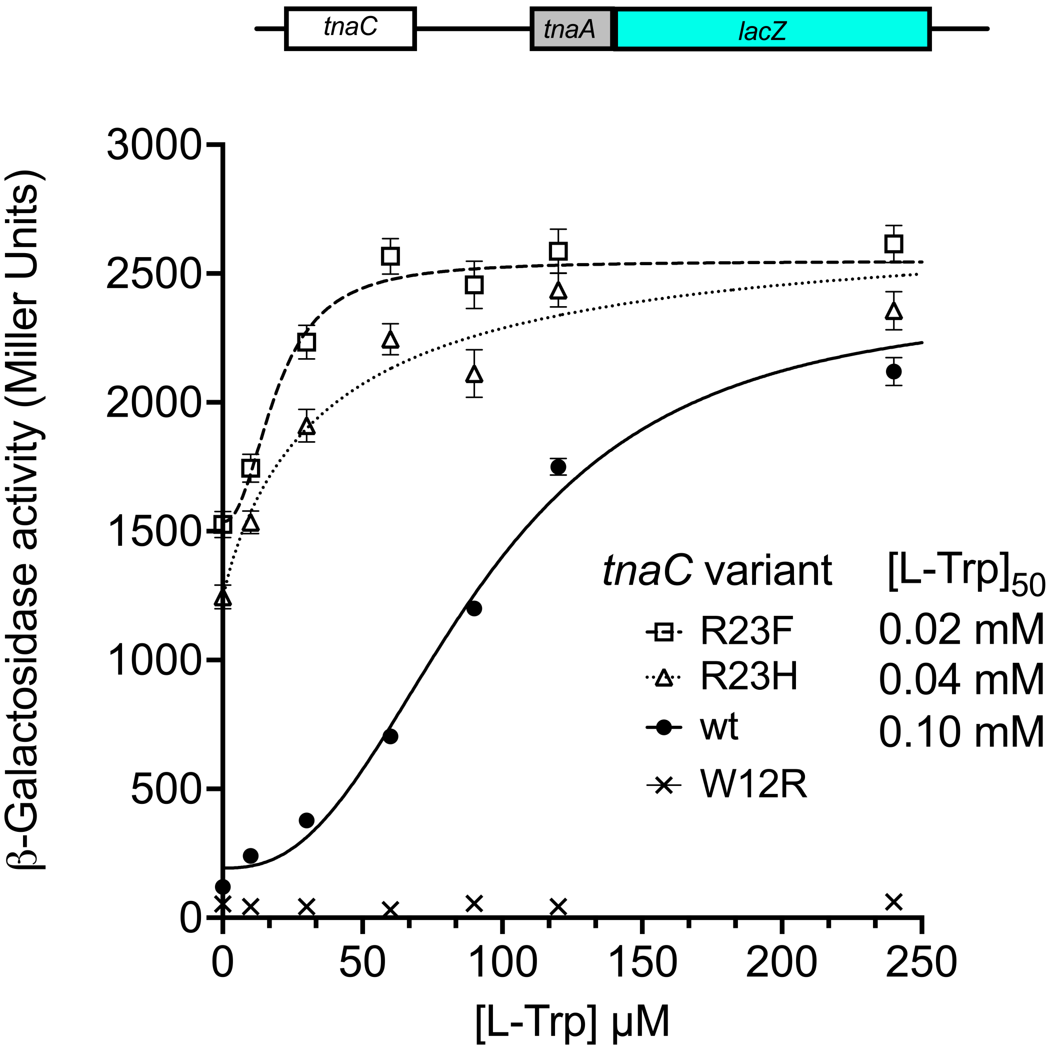


**Figure S1 | R23F and R23H mutations increase the sensitivity of an inducible *tnaC-tnaA-lacZ* reporter gene to L-Trp.** Bacterial cultures of strains with *tnaC-tnaA-lacZ* reporter genes (structure shown above) containing the indicated *tnaC* variants were grown in minimal media at 37ºC in the presence of several concentrations of L-Trp until they reached $OD_{600}$ = 0.6. Cells were then processed as previously indicated to determine the expression of *lacZ* (Ref. 16). Bars indicate the standard error to mean value for each point taken (some of the error bars are too narrow to be seen). Each plot represents three independent experiments. $[L\text{-}Trp]_{50}$ values were calculated using a non-linear fit model where each plot has a coefficient of determination above 0.96.

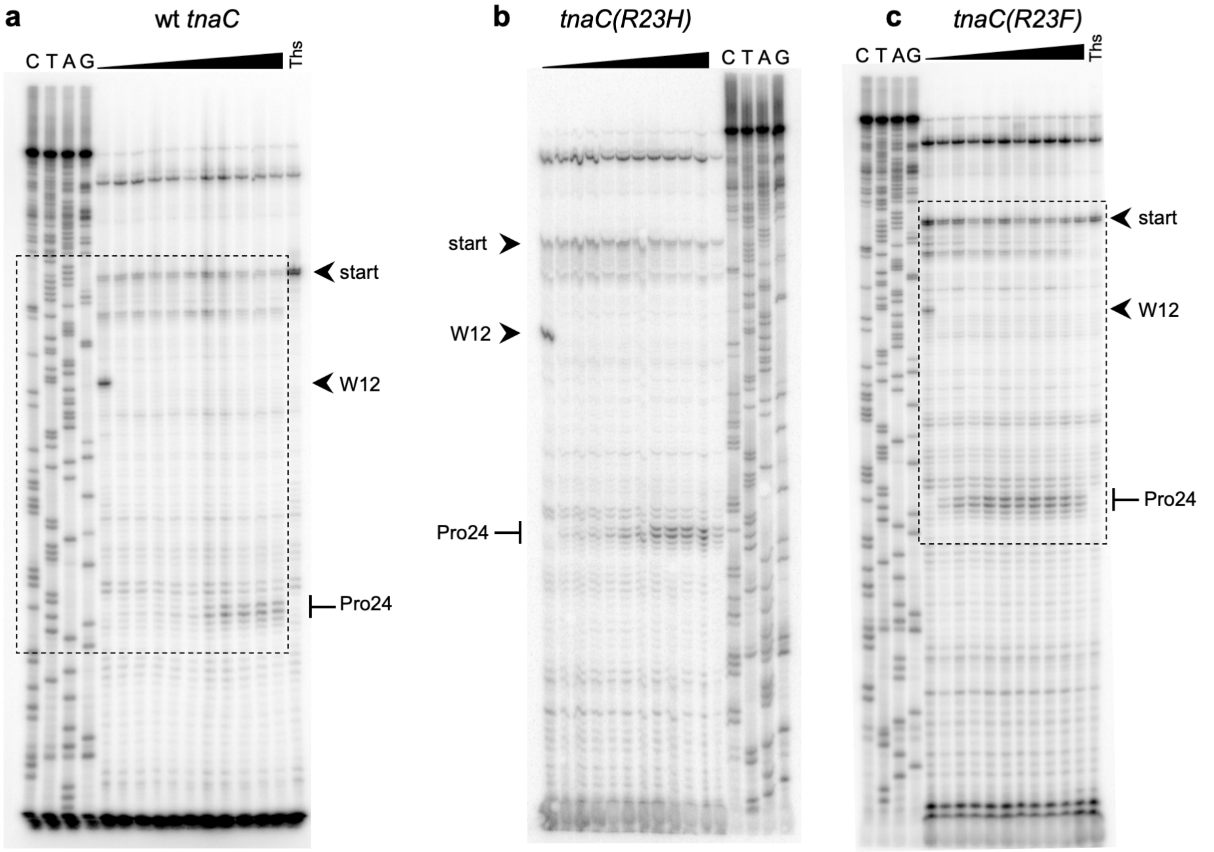


**Figure S2 | Determination of ribosome arrest by toeprinting assays of *tnaC* variants over a range of L-Trp concentrations.** L-Trp concentrations ranging from 0 to 10 mM (indicated by the gradient symbol) were used in these assays. Arrested ribosome signals at the start codon (start), Trp12 (W12) and Pro24 codons are indicated in each gel. Samples treated with thiostrepton (Ths) were used as controls to differentiate between signals produced by arrested ribosomes at the *tnaC* ORF and signals that are not produced by translating ribosomes. Gels in panels (a) and (c) are the entire gels that were cropped (dotted line box) in Figure 1b. These results show that the R23F mutation (c) increases the sensitivity of ribosome stalling to L-Trp to a greater extent than the R23H mutation (b). Each of the three gels is representative of n=3 independent experiments.

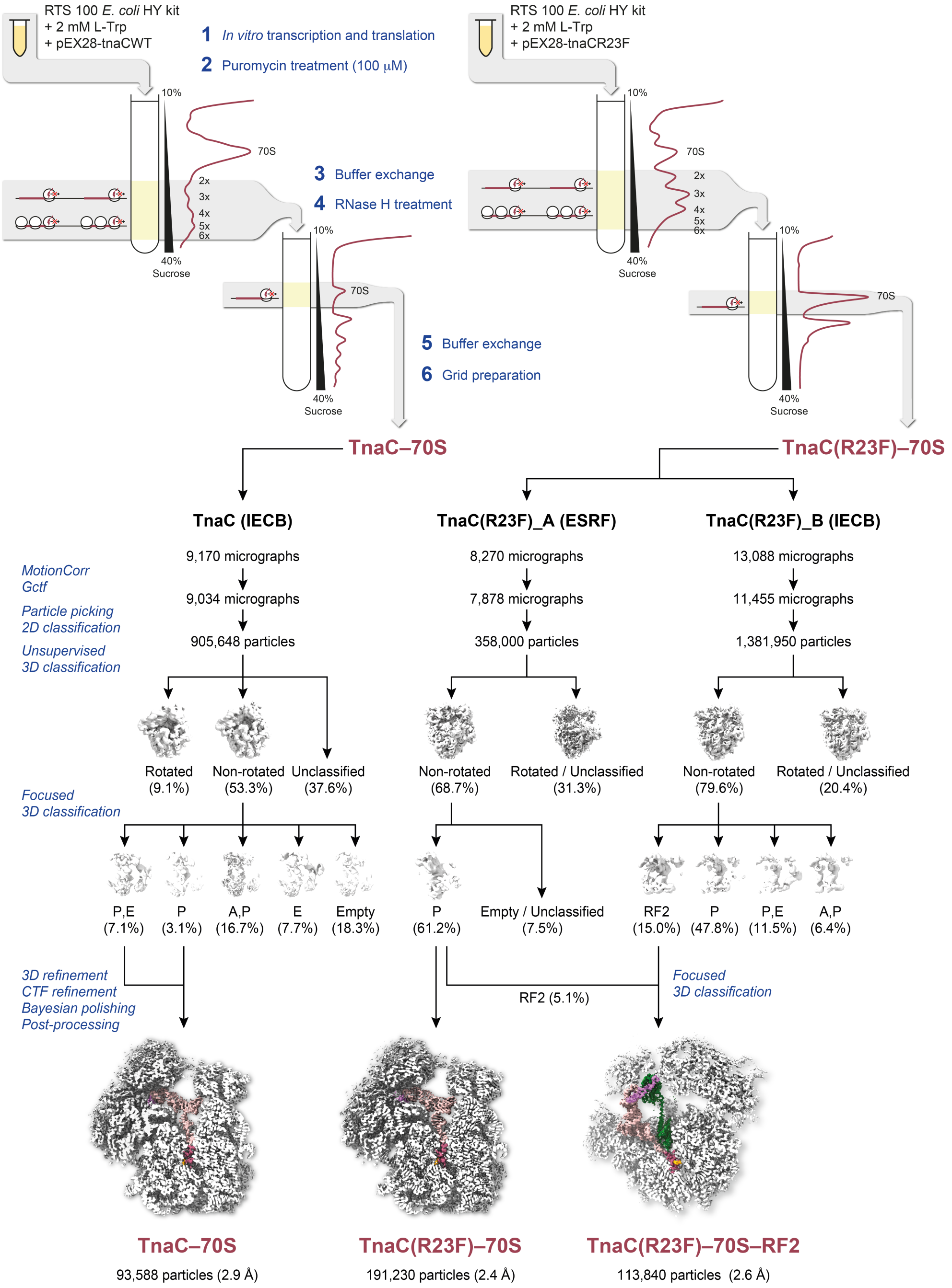


**Figure S3 | Complex purification and cryo-EM data processing workflow.** Stalled ribosomal complexes were prepared using a bicistronic operon containing two identical copies of *tnaC* or *tnaC(R23F)* (Supplementary Table 3). A first sucrose gradient was performed to collect polysomes, followed by a second sucrose gradient after RNase H treatment to collect the monosomal fraction, which was then used to prepare the grids for cryo-EM data acquisition. The flowchart shows the workflow used to enrich the stalled complexes of interest, and to process and analyze the resulting cryo-EM data. Cross-sections of the final reconstructions are shown with the 70S ribosome in white, the P-site tRNA in pink, the mRNA in dark pink, the TnaC peptide in red and the L-Trp ligand in orange.

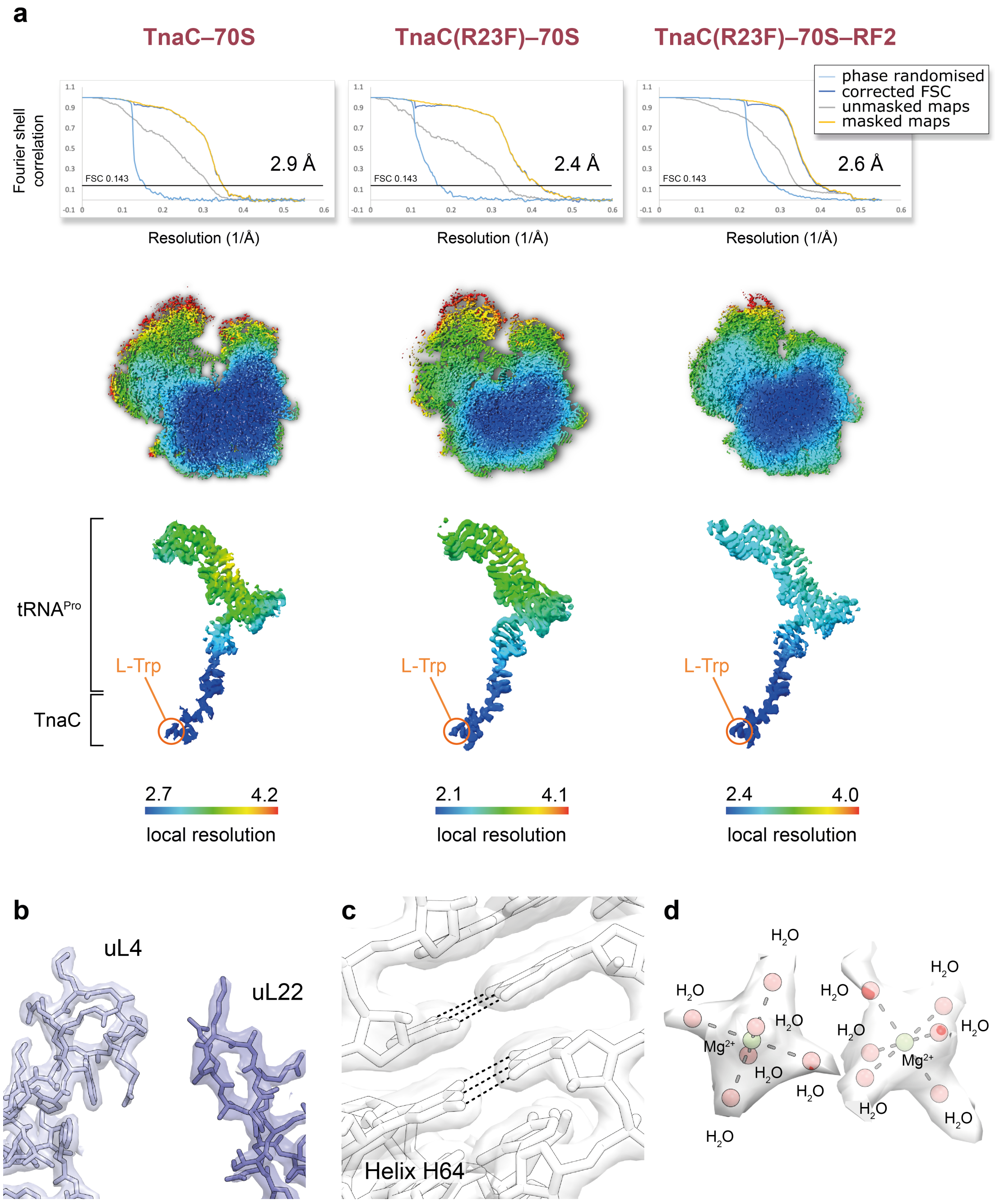


**Figure S4 | Quality of the cryo-EM reconstructions**. (a) Fourier Shell Correlation (FSC) curves of the final cryo-EM reconstructions are shown as calculated by the *RELION 3.1* (Ref. 51) post-processing algorithm. Cross-sections of the TnaC–70S, TnaC(R23F)–70S and TnaC(R23F)–70S–RF2 maps and segmented densities for TnaC-tRNA$^{Pro}$ and L-Trp are colored according to local resolution calculated by *RELION 3.1* (Ref. 51). Details of the TnaC(R23F)–70S map showing (b) the density and fitted atomic models for ribosomal proteins uL4 (light blue) and uL22 (periwinkle blue), near the ribosomal exit tunnel, (c) helix 64 of the 23S large subunit, and (d) two adjacent hydrated magnesium ion clusters in the core of the ribosomal large subunit.

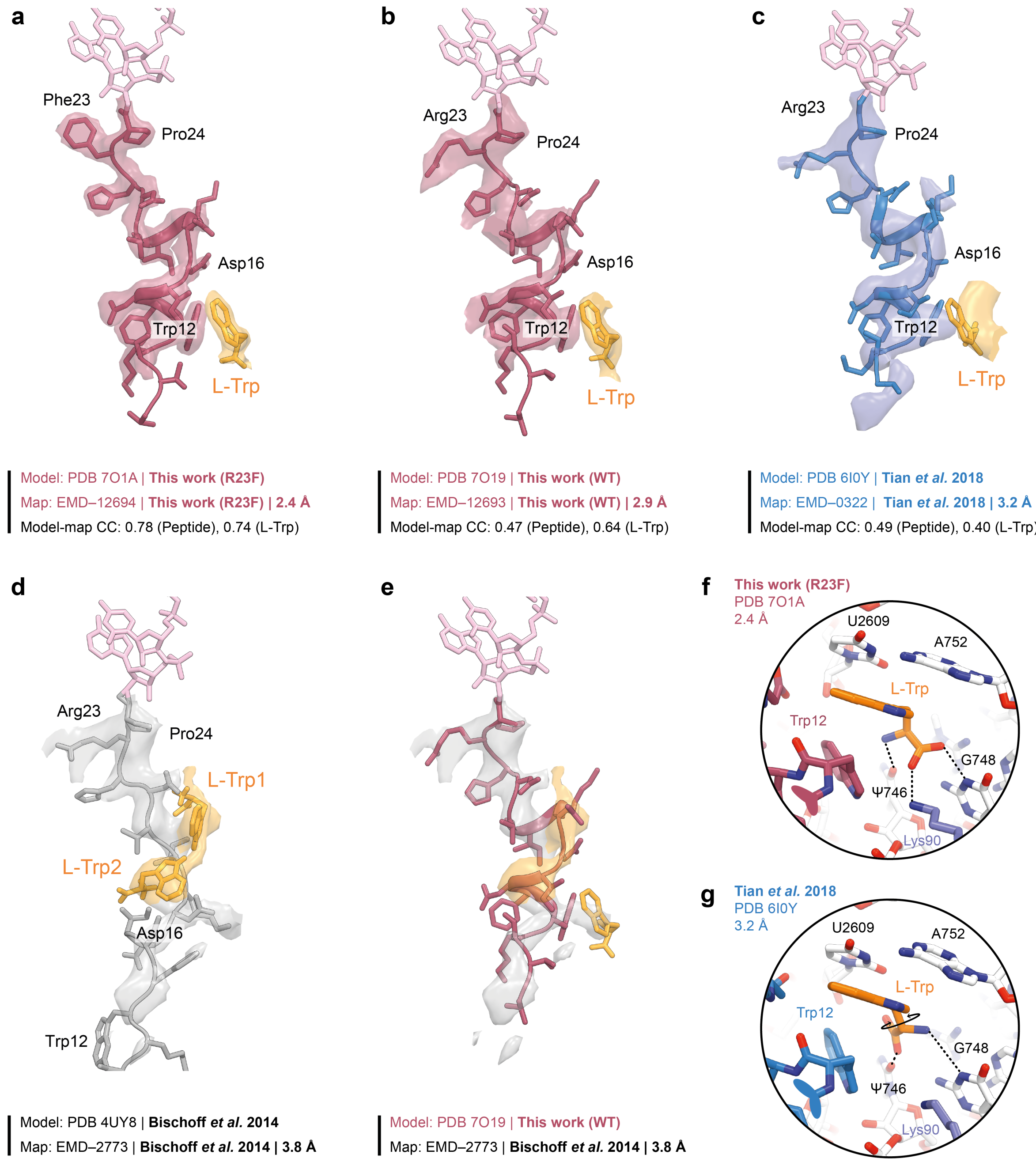


**Figure S5 | Comparison of the structures from this study with earlier models**. Segmented cryo-EM densities and fitted models for the nascent peptide (red, blue or gray) and L-Trp ligand (orange) are shown for (a) TnaC(R23F)–70S, (b) TnaC–70S, (c) a ribosome stalled during translation of a titin I27-TnaC chimera (Ref. 22), and (d) an earlier 3.8 Å cryo-EM reconstruction of a stalled TnaC–ribosome complex thought to have contained two bound L-Trp molecules (Ref. 21). (e) Atomic model of wild-type TnaC obtained in this study fitted into the density from the 3.8 Å structure by Bischoff *et al.* (Ref. 21). Atomic models of the lone L-Trp ligand within its binding pocket in the (f) TnaC(R23F)–70S (red) and (g) titin I27-TnaC chimera (blue, ref. 22) structures. Note the missing salt bridge between the carboxylic acid of L-Trp the side chain amine of Lys90 in the titin I27-TnaC chimera. Segmented densities for TnaC and L-Trp are shown using the same contour level.

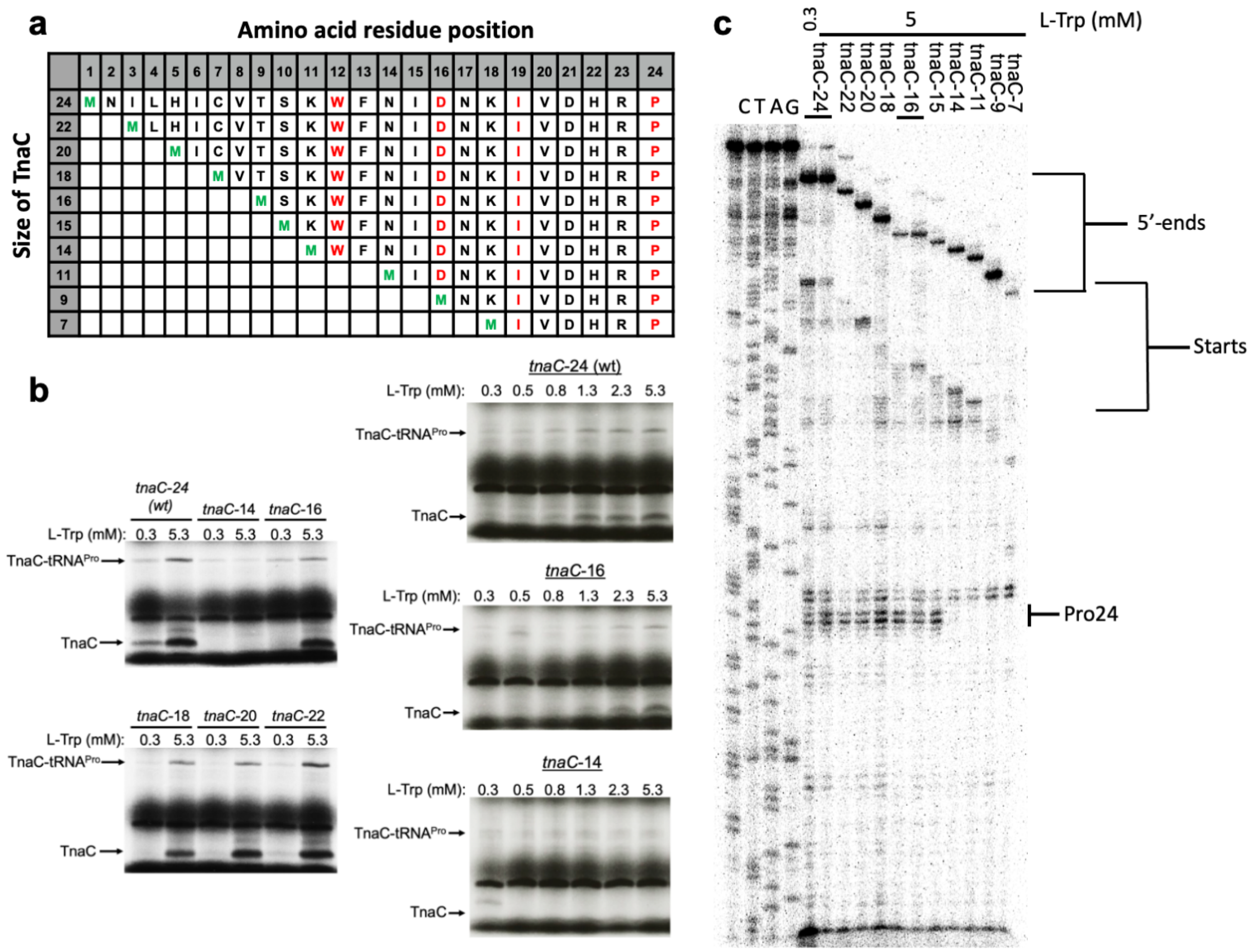


**Figure S6 | Effect of N-terminal deletions on the ability of TnaC to undergo translational arrest in the presence of L-Trp.** (a) Diagram representing the sequences of the TnaC peptides used in the experiments shown in b and c. Green letters indicate the first decoded amino acid; red letters indicate conserved TnaC residues that have been shown experimentally to be functionally important. (b) Radiograms showing *in vitro* translation reactions performed as indicated before (Ref. 16). (c) Radiogram of a toe-printing assay performed as indicated before (Ref. 16). Each of the gels is representative of n=3 independent experiments.

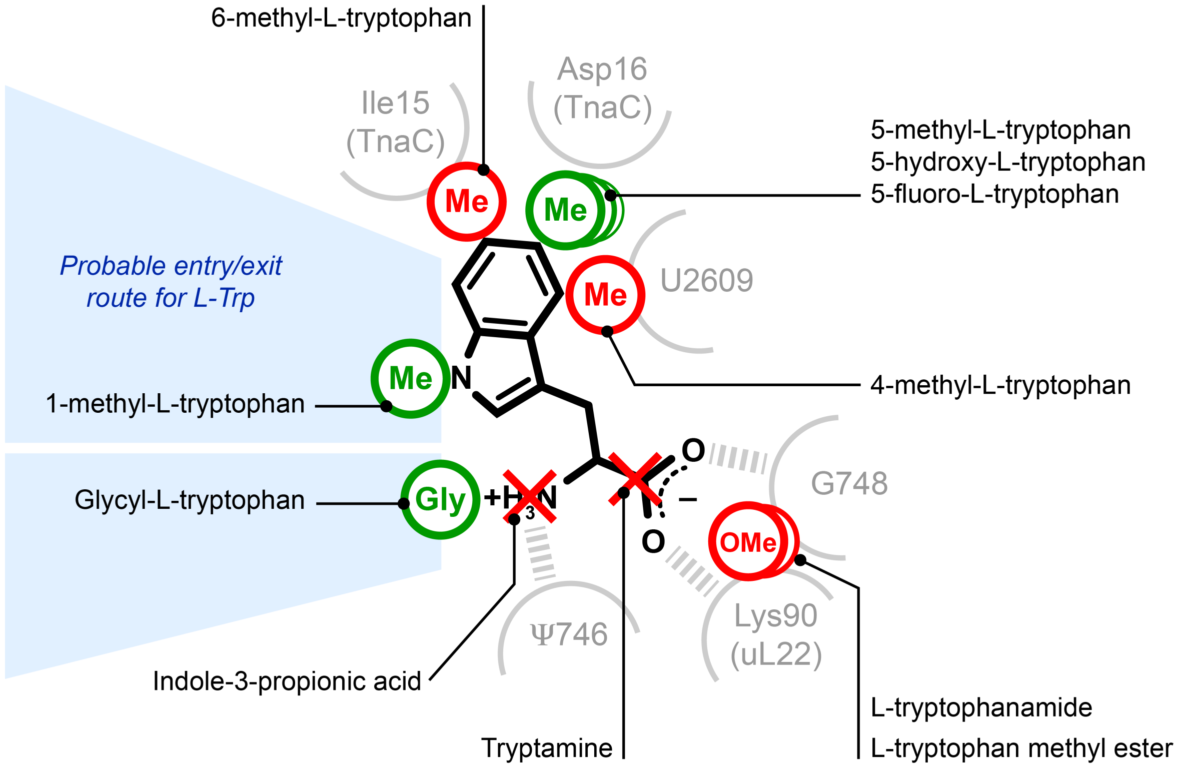


**Figure S7 | Effect of ligand modifications on the ability of TnaC to undergo translational arrest.** Ligand modifications (Ref. 26) that abolish stalling are shown in red, while neutral modifications are shown in green. Solvent channels adjacent to the L-Trp molecule, including a probable entry/exit route for this ligand, are shown in light blue.

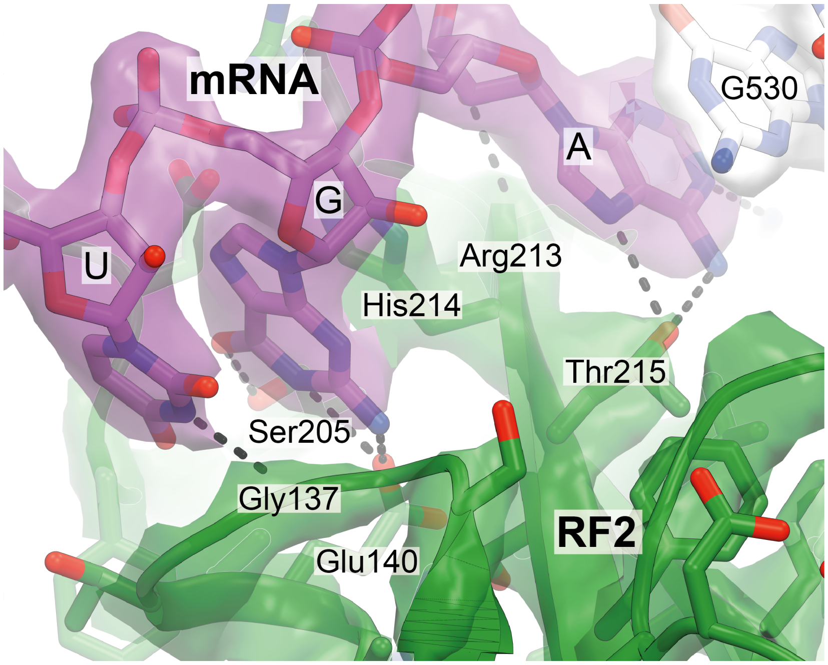


**Figure S8 | Interactions between RF2 and the UGA stop codon in the TnaC(R23F)–70S–RF2 structure.** The stop codon in the A-site (purple) is recognized by domain II of RF2 (green). Segmented densities for the ribosome, the mRNA and RF2 are shown using the same contour level.
