# Supplementary material for: Structural basis for the tryptophan sensitivity of TnaC-mediated ribosome stalling
Source: Nat Commun. 2021 Sep 9;12:5340. doi: 10.1038/s41467-021-25663-8 (PMC8429421; doi:10.1038/s41467-021-25663-8)
Supplement: Supplementary file 3 — Reporting Summary [file 41467_2021_25663_MOESM3_ESM.pdf]

nature research

Corresponding author(s): Innis, Cruz-Vera, Sachs

Last updated by author(s): Aug 19, 2021

Reporting Summary

Nature Research wishes to improve the reproducibility of the work that we publish. This form provides structure for consistency and transparency in reporting. For further information on Nature Research policies, see our Editorial Policies and the Editorial Policy Checklist.

Statistics

For all statistical analyses, confirm that the following items are present in the figure legend, table legend, main text, or Methods section.

n/a	Confirmed	
☐	☒	The exact sample size (*n*) for each experimental group/condition, given as a discrete number and unit of measurement
☐	☒	A statement on whether measurements were taken from distinct samples or whether the same sample was measured repeatedly
☐	☒	The statistical test(s) used AND whether they are one- or two-sided<br>*Only common tests should be described solely by name; describe more complex techniques in the Methods section.*
☐	☒	A description of all covariates tested
☐	☒	A description of any assumptions or corrections, such as tests of normality and adjustment for multiple comparisons
☐	☒	A full description of the statistical parameters including central tendency (e.g. means) or other basic estimates (e.g. regression coefficient) AND variation (e.g. standard deviation) or associated estimates of uncertainty (e.g. confidence intervals)
☐	☒	For null hypothesis testing, the test statistic (e.g. *F*, *t*, *r*) with confidence intervals, effect sizes, degrees of freedom and *P* value noted<br>*Give P values as exact values whenever suitable.*
☒	☐	For Bayesian analysis, information on the choice of priors and Markov chain Monte Carlo settings
☒	☐	For hierarchical and complex designs, identification of the appropriate level for tests and full reporting of outcomes
☒	☐	Estimates of effect sizes (e.g. Cohen's *d*, Pearson's *r*), indicating how they were calculated

Our web collection on statistics for biologists contains articles on many of the points above.

Software and code

Policy information about availability of computer code

Data collection	Commercial software used in this work includes EPU 2.8.1 and GraphPad Prism 9
Data analysis	Open source software used for data analysis in this work includes Fiji ImageJ 2.1.0/1.53c, SerialEM 3.8.0, Relion 3.1-beta (MotionCor2 and Gctf as implemented in the Relion 3.1-beta release), Phenix 1.19.2 (including douse, which is part of this release), Coot 0.8.1, Isolde 1.1, OpenSource Pymol 1.7.4.0, ChimeraX 1.1.1

For manuscripts utilizing custom algorithms or software that are central to the research but not yet described in published literature, software must be made available to editors and reviewers. We strongly encourage code deposition in a community repository (e.g. GitHub). See the Nature Research guidelines for submitting code & software for further information.

Data

Policy information about availability of data

All manuscripts must include a data availability statement. This statement should provide the following information, where applicable:

- Accession codes, unique identifiers, or web links for publicly available datasets
- A list of figures that have associated raw data
- A description of any restrictions on data availability

The TnaC–70S, TnaC(R23F)–70S and TnaC(R23F)–70S–RF2 structures obtained in this study have been deposited with the Research Collaboratory for Structural Bioinformatics Protein Data Bank under accession codes 7O19 [https://doi.org/10.2210/pdb7O19/pdb], 7O1A [https://doi.org/10.2210/pdb7O1A/pdb] and 7O1C [https://doi.org/10.2210/pdb7O1C/pdb]; the cryo-EM maps generated in this study have been deposited with the Electron Microscopy Data Bank under accession codes EMD-12693 [https://www.ebi.ac.uk/emdb/entry/EMD-12693], EMD-12694 [https://www.ebi.ac.uk/emdb/entry/EMD-12694] and EMD-12695 [https://www.ebi.ac.uk/emdb/entry/EMD-12695]. Raw movie stacks generated in this study have been deposited with the Electron Microscopy Public Image Archive under accession code EMPIAR-10695 [https://www.ebi.ac.uk/empiar/EMPIAR-10695/]. Data for Fig. 1c, 1f and Supplementary Fig. 1 are provided in the Source Data file.

Field-specific reporting

Please select the one below that is the best fit for your research. If you are not sure, read the appropriate sections before making your selection.

- [x] Life sciences
- [ ] Behavioural & social sciences
- [ ] Ecological, evolutionary & environmental sciences

For a reference copy of the document with all sections, see nature.com/documents/nr-reporting-summary-flat.pdf

Life sciences study design

All studies must disclose on these points even when the disclosure is negative.

Sample size	No statistical methods were used to predetermine sample size. Sample sizes for this qualitative study (n=3) were a compromise between experimental cost and being able to generate standard error of the mean (SEM) values.
Data exclusions	No data were excluded from the analyses.
Replication	All biochemical assays were performed in triplicate. All attempts at replication were successful.
Randomization	The experiments were not randomized. Randomization is not relevant to this study, since the outcomes of these experiments are not dependent on the judgment of the researcher and depend on control reactions that cannot be affected by unconscious bias.
Blinding	Investigators were not blinded to allocation during experiments and outcome assessment. Blinding is not relevant to this study, since the outcomes of these experiments are not dependent on the judgment of the researcher and depend on control reactions that cannot be affected by unconscious bias.

Reporting for specific materials, systems and methods

We require information from authors about some types of materials, experimental systems and methods used in many studies. Here, indicate whether each material, system or method listed is relevant to your study. If you are not sure if a list item applies to your research, read the appropriate section before selecting a response.

Materials & experimental systems

n/a	Involved in the study	
[ ]	[x]	Antibodies
[x]	[ ]	Eukaryotic cell lines
[x]	[ ]	Palaeontology and archaeology
[x]	[ ]	Animals and other organisms
[x]	[ ]	Human research participants
[x]	[ ]	Clinical data
[x]	[ ]	Dual use research of concern

Methods

n/a	Involved in the study	
[x]	[ ]	ChIP-seq
[x]	[ ]	Flow cytometry
[x]	[ ]	MRI-based neuroimaging

Antibodies

Antibodies used	Polyclonal serum obtained from rabbits.
Validation	Yes, using pure antigen RF2 under western blot conditions. Dilutions 1:10000 of the serum was used to detect a range between 1-20 pg of antigen.
